# Implementation of a low-titre whole blood transfusion program in a civilian helicopter emergency medical service

**DOI:** 10.1186/s13049-022-01051-z

**Published:** 2022-12-09

**Authors:** Geir Arne Sunde, Christopher Bjerkvig, Marit Bekkevold, Einar K. Kristoffersen, Geir Strandenes, Øyvind Bruserud, Torunn Oveland Apelseth, Jon-Kenneth Heltne

**Affiliations:** 1 Department of Anesthesia and Intensive Care, Haukeland University Hospital, Bergen, Norway; 2 Helicopter Emergency Medical Services, Bergen, Norway; 3 Department of Research, Norwegian Air Ambulance Foundation, Oslo, Norway; 4 Division of Prehospital Services, Air Ambulance Department, Oslo University Hospital, Oslo, Norway; 5 Department of Clinical Medicine, Faculty of Medicine, University of Bergen, Bergen, Norway; 6 Department of Clinical Science, Faculty of Medicine, University of Bergen, Bergen, Norway; 7 Department of Immunology and Transfusion Medicine, Haukeland University Hospital, Bergen, Norway; 8 Norwegian Armed Forces Joint Medical Service, Sessvollmoen, Norway

**Keywords:** Out of hospital, Blood transfusion, Haemorrhagic shock, Helicopter Emergency Medical Services, Low-titre group O whole blood

## Abstract

**Background:**

Early balanced transfusion is associated with improved outcome in haemorrhagic shock patients. This study describes the implementation and evaluates the safety of a whole blood transfusion program in a civilian helicopter emergency medical service (HEMS).

**Methods:**

This prospective observational study was performed over a 5-year period at HEMS-Bergen, Norway. Patients in haemorrhagic shock receiving out of hospital transfusion of low-titre Group O whole blood (LTOWB) or other blood components were included. Two LTOWB units were produced weekly and rotated to the HEMS for forward storage. The primary endpoints were the number of patients transfused, mechanisms of injury/illness, adverse events and survival rates. Informed consent covered patient pathway from time of emergency interventions to last endpoint and subsequent data handling/storage.

**Results:**

The HEMS responded to 5124 patients. Seventy-two (1.4%) patients received transfusions. Twenty patients (28%) were excluded due to lack of consent (16) or not meeting the inclusion criteria (4). Of the 52 (100%) patients, 48 (92%) received LTOWB, nine (17%) received packed red blood cells (PRBC), and nine (17%) received freeze-dried plasma. Of the forty-six (88%) patients admitted alive to hospital, 35 (76%) received additional blood transfusions during the first 24 h. Categories were blunt trauma 30 (58%), penetrating trauma 7 (13%), and nontrauma 15 (29%). The majority (79%) were male, with a median age of 49 (IQR 27–70) years. No transfusion reactions, serious complications or logistical challenges were reported. Overall, 36 (69%) patients survived 24 h, and 28 (54%) survived 30 days.

**Conclusions:**

Implementing a whole blood transfusion program in civilian HEMS is feasible and safe and the logistics around out of hospital whole blood transfusions are manageable.

*Trial registration* The study is registered in the ClinicalTrials.gov registry (NCT02784951).

**Supplementary Information:**

The online version contains supplementary material available at 10.1186/s13049-022-01051-z.

## Introduction

Haemorrhage is a leading cause of early death in trauma patients worldwide, but the optimal haemorrhagic shock resuscitation strategy is still under discussion [1, 2]. Development in military and civilian trauma care over the last decade indicates that crystalloid or colloid-based resuscitation causes haemodilution, acidosis and reduced oxygen delivery, suggesting that an early blood-based resuscitation strategy can be superior for treating major haemorrhage in the out of hospital setting [3, 4]. Military use of blood products is a key element in remote damage control resuscitation (RDCR), as it reduces the time to transfusion and improves survival in combat causalities [5].

Recently, some civilian emergency medical services have established systems for whole blood, packed red blood cells (PRBC) and plasma administration [6, 7]. Whole blood shows logistical advantages over a balanced transfusion strategy using PRBC and plasma, is less haemodiluted and contains platelets that improve clotting [8, 9]. Whole blood transfusions may improve shock severity and coagulopathy compared to traditional resuscitation with crystalloid fluids or blood components [10]. Improved survival in civilian trauma patients receiving whole blood in the emergency department has been shown [9].

However, evidence describing out of hospital whole blood use in civilian haemorrhagic shock patients is scarce. In 2015, our helicopter emergency medical service (HEMS) was one of the first civilian physician-staffed services to implement an out of hospital whole blood transfusion program. This study aimed to describe and evaluate the implementation and safety of this transfusion program, with primary endpoints being the number and category of patients transfused, transfusion-related adverse events and 24-h/30-day survival rates.

## Methods

### Ethical approval

The Regional Committee for Medical and Health Research Ethics in Norway (REK-Vest-2016/304) approved the study. Informed consent covered patient pathway from time of emergency interventions to last endpoint and subsequent data handling/storage. Written informed consent was obtained from surviving subjects and waived for deceased participants. The study is registered in the ClinicalTrials.gov registry (NCT02784951).

### Patients

Patients in haemorrhagic shock and receiving out of hospital transfusion of low-titre Group O whole blood (LTOWB) or PRBC were included. Transfusions were done in compliance with HEMS standard operating procedures describing the use of blood products and the accompanying bundle of care (e.g., tranexamic acid, calcium or pelvic binders). Transfusion was initiated at the physician’s discretion in patients with a mechanism of injury or illness compatible with active bleeding and in haemorrhagic shock (e.g., penetrating trauma to the torso), absent or elevated radial pulse (above 100 beats per minute), decreased systolic blood pressure (SBP) below 90 mmHg, or altered mental status (reduced Glasgow Coma Scale (GCS)) in the absence of head injury or known intoxication. Exclusion criteria were previous allergic reactions to blood transfusions or refusal of blood products on religious grounds (e.g., Jehovah’s Witness).

### Conduct of the study

This prospective observational study was performed at HEMS-Bergen over a 5-year period from December 2015 to December 2020. The HEMS is located at the regional trauma centre for the western part of Norway at Haukeland University Hospital in Bergen and is staffed by a physician, flight paramedic, and pilot. All physicians are specialists in anaesthesiology, with a median HEMS experience of 16.5 (IQR 6–19.5) years. The pilots have extensive military or civilian flight experience prior to starting HEMS service, with all-weather/instrument flight/night-vision-goggles capabilities. The flight paramedics are paramedics or nurses, and fulfil national standards for HEMS flight paramedics issued by the Ministry of Justice and Public Security (e.g., education, rescue and medical competence). The HEMS covers a population of approximately 550,000 people across 15,500 km^2^, including urban and rural areas, a coastline, several fjords, and high mountains. It operates 24/7/365 and responds by helicopter or rapid response car to medical and trauma cases (ratio 60:40). Data were obtained from patient records. Out of hospital injury severity was classified using the National Advisory Committee on Aeronautics’ (NACA) severity score [11]. The study complied with Strengthening the Reporting of Observational studies in Epidemiology (STROBE) reporting guidelines [12].

### Endpoints

Primary endpoints were the number and category of patients transfused, transfusion-related adverse events and 24-h/30-day survival rates. Secondary endpoints were key vital signs, HEMS response times and emergency interventions.

### Blood products and logistics

LTOWB units are produced weekly at the Department of Immunology and Transfusion Medicine (Haukeland University Hospital, Bergen) and rotated to the HEMS for forward storage at the base [13]. If unused, the units are rotated back and used for a total storage period of up to 21 days. If transfused, they are immediately replaced with LTOWB or freshly produced PRBC (0 Rh (D) negative) units. The LTOWB units are donated by regular blood donors and tested for infectious agents and blood type antibodies in accordance with Norwegian and European legal regulations [14]. The LTOWB is leukocyte reduced with a platelet-sparing filter (IMUFLEX, WB-SP Blood Bag System, TERUMOBCT, Lakewood, CO, USA). Continuous quality monitoring of used blood products is performed at the Department of Immunology of Transfusion Medicine in accordance with the European recommendations [14]. If needed, additional transfusion with freeze-dried plasma (FDP) (LyoPlas N-w (Deutsches Rotes Kreuz—Blutspendedienst West, Hagen, Germany) may be given. The FDP used is a quarantined single donor plasma. We currently use group AB or A plasma. LyoPlas powder dissolves in 200 ml of sterile water and is ready for injection within 5–10 min depending on water temperature and can be administered intravenously or intraosseously.

The HEMS is deployed with two units of LTOWB and one unit of FDP. The HEMS crew, including flight paramedics and pilots, are trained in administering transfusions of LTOWB, FDP or PRBC on the physician’s orders. The LTOWB units are stored in a portable thermal “golden hour box” (Pelican Biothermal, Plymouth, MN, USA). Crēdo Duracube, Series 4 2L HD, 2–8 °C, 272), and the temperature is monitored with a temperature logger. The box is stored in a refrigerator at the HEMS base at 4 °C between missions. We have validated the box to maintain temperature at 4 °C under these conditions for more than a week [7].

### Statistics

Continuous variables are expressed as medians with interquartile ranges (IQRs) or means (standard deviations), and categorical variables are expressed as numbers with percentages of the total. The Wilcoxon matched-pairs signed rank test was used when comparing out of hospital and in-hospital vital signs. *P* values less than 0.05 were considered statistically significant. The statistical package for IBM SPSS statistics (SPSS, version 26.0, IBM Corporation, New York, USA) was used for statistical analysis.

## Results

### Patients

**Fig. 1:**
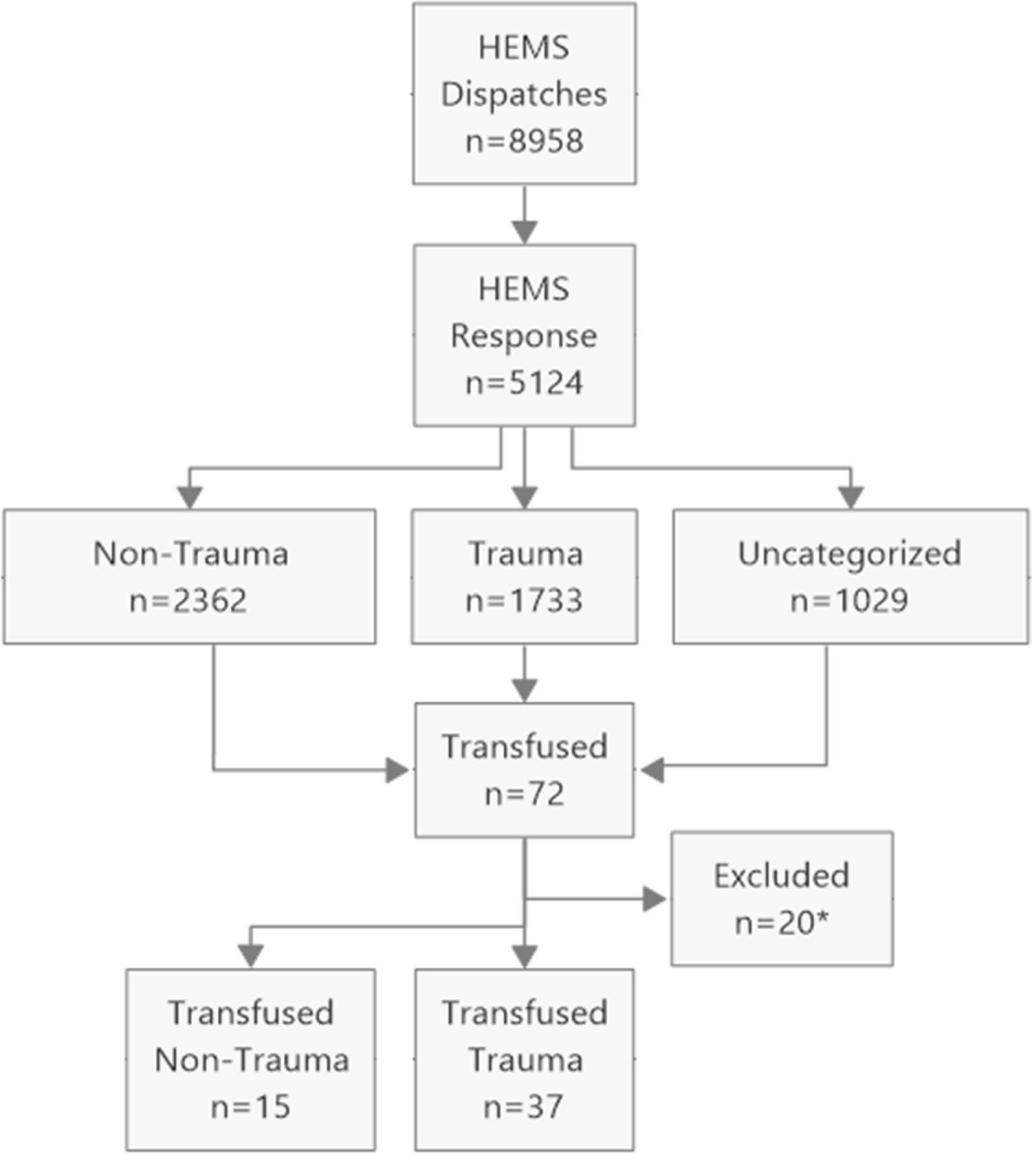
The flowchart shows the total number of HEMS dispatches and the number of responses by category in the period from December 2015 to December 2020. In total, 72 patients were transfused. *Sixteen patients were excluded due to lack of informed consent, and 4 patients were excluded since they did not meet the inclusion criteria.

In the 5-year study period, the HEMS responded to 5124 patients. Seventy-two patients received out of hospital LTOWB, FDP or PRBC transfusions from December 2015 to December 2020. Sixteen patients were excluded due to lack of consent, and four patients did not meet the inclusion criteria. We analysed data from the remaining 52 (100%) patients with written consent (Fig. 1: flowchart). The mean NACA score was 5.63 (SD 0.864) (Additional file 1). Patient characteristics are summarized in Table 1, and biochemistry analyses are summarized in Table 2. The main mechanisms of injury in trauma patients were motor vehicle accidents (26.9%) and falls from heights (15.4%). In nontrauma patients, gastrointestinal bleeding (15.4%) and ruptured aortae (9.6%) dominated (Table 3). The overall transfusion rate was 1.4%.

**Table 1:** Basic characteristics

Variable	All cases	Trauma	Nontrauma
No. of patients	52 (100)	37 (100)	15 (100)
Male	41 (79)	28 (76)	13 (87)
Age, y	49 (27–70)	41 (21–62)	66 (54–74)
*Mission category*			
Primary	43 (83)	33 (89)	10 (67)
Secondary	9 (17)	4 (11)	5 (33)
*Transport mode*			
Ground ambulance	27 (55)	17 (46)	10 (67)
Helicopter	20 (41)	15 (41)	5 (33)
Not transported	2 (4)	2 (5)	0 (0)
*NACA score*	6 (5–6)	6 (5–6)	6 (5–6)
*Timeline*			
Response time, min	21 (13–40)	21 (12–37)	25 (14–40)
On-scene time, min	10 (4–20)	11 (4–22)	8 (4–10)
On-scene to hospital arrival, min	20 (13–30)	19 (13–27)	20 (13–36)
*Type of injury*			
Trauma	37 (71)	37 (100)	0 (0)
Nontrauma	15 (29)	0 (0)	15 (100)
*Out of hospital* *Blood pressure assessment*			
Not measured	5 (10)	4 (11)	1 (7)
Central palpable pulse	9 (17)	6 (16)	4 (27)
Peripheral palpable pulse	3 (6)	3 (8)	0 (0)
Measured blood pressure	24 (46)	15 (41)	9 (60)
Cardiac arrest	10 (19)	9 (24)	1 (7)
*Airway management*			
Endotracheal intubation	15 (29)	13 (35)	2 (13)
Supraglottic device	3 (6)	3 (8)	0 (0)
Mask-bag ventilation	4 (8)	4 (11)	0 (0)
Supplementary oxygen	17 (33)	12 (32)	5 (33)
No airway intervention	13 (25)	5 (14)	8 (53)
*Out of hospital*			
Systolic blood pressure, mmHg	80 (0–100)	75 (0–98)	97 (50–100)
Heart rate, beats/min	80 (0–100)	65 (0–90)	120 (94–125)
Respiratory rate, breaths/min	13 (0–20)	10 (0–17)	16 (12–28)
Pulse oximetry, %	90.5 (0–99)	84 (0–99)	94 (88–100)
Glasgow coma scale score	9.5 (3–15)	8 (3–15)	12 (3–15)
*In-hospital*			
*Emergency department*			
Systolic blood pressure, mmHg	110 (65–130)	110 (76–130)	116 (60–126)
Heart rate, beats/min	94 (70–108)	84 (64–106)	100 (82–113)
Respiratory rate, breaths/min	20 (12–24)	15 (12–24)	22 (16–27)
Pulse oximetry, %	97 (92–100)	98 (88–100)	97 (93–100)
Glasgow coma scale score	12 (3–15)	7 (3–15)	15 (10–15)
*Interventions*			
Emergency interventions < 24 h	41 (79)	29 (78)	12 (80)
*Survival*			
Survival 24 h	36 (69)	25 (68)	11 (73)
Survival 30 days	28 (54)	18 (49)	10 (67)

Data are presented as the number of patients (percentage) or median (interquartile range) per patient category

NACA score, National Advisory Committee for Aeronautics score

**Table 2:** First biochemistry analyses in-hospital (N = 52)

Patients transfused	Number of patients (%)	Values
Haemoglobin (g/dl)	42 (80)	12.9 (11.5–14.1)
Haematocrit	4 (7)	0.30 (0.3–0.4)
INR	25 (48)	1.1 (1.1–1.2)
APTT	24 (46)	33.5 (31–39)
Fibrinogen (g/L)	23 (44)	2.4 (1.8–3.1)
pH	39 (75)	7.3 (7.2–7.4)
Lactate (mmol/L)	39 (75)	4.4 (2.0–10.1)
Base excess	39 (75)	− 5.7 (− 15.7–(− 1.1))
paO_2_	39 (75)	22.7 (10.8–36.6)
paCO_2_	39 (75)	5.4 (4.3–6.4)
Ionized calcium	38 (73)	1.10 (1.0–1.2)

Data are presented as the number of patients (percentage) or median (interquartile range)

APTT, activated partial thromboplastin time; INR, international normalized ratio

**Table 3:** Mechanisms of injury or illness

Mechanisms of injury or illness	Number of patients	%
Avalanche	1	1.9
Cardiac tamponade	1	1.9
Chainsaw injury	1	1.9
Extremity injury	1	1.9
Epistaxis	1	1.9
Fall from heights- multitrauma	8	15.4
Gastrointestinal bleeding	8	15.4
Gunshot wounds- multitrauma	2	3.8
Motor vehicle accident- multitrauma	14	26.9
Ruptured aorta	5	9.6
Stab injuries- multitrauma	4	7.7
Surgical complications	1	1.9
Traumatic brain injury	1	1.9
Vehicle versus pedestrian accident	2	3.8
Wind-thrown tree	2	3.8
Total	52	100.0

### Survival

Overall, 36 (69%) patients survived the first 24 h, and 28 (54%) patients survived 30 days. Of the 24 (46%) deaths in total, six (12%) died within 1 h, an additional seven (13%) within 6 h and three (6%) within 24 h. The remaining eight (15%) died within 30 days. Ten (19%) patients presented with cardiac arrest (CA) on-scene: seven (70%) with blunt trauma, two (20%) with penetrating trauma and one (10%) with nontrauma. Four (40%) patients with initial CA on-scene were admitted with signs of life. However, no patients presenting with CA on-scene survived beyond 24 h.

### Transfusion-related adverse events and waste

No clinical transfusion reactions were reported out of hospital or after admission. Furthermore, there were no other serious complications or adverse events related to the transfusion procedure. Each blood unit in the blood bank inventory is electronically supervised from donor to patient by a single blood bank laboratory information system, including those at the HEMS base. No waste of prepared plasma or blood units was recorded at the HEMS due to rotation of the unused units to the in-hospital use at the Department of Immunology and Transfusion Medicine. In 47 (90%) patients, the physicians reported no technical problems with the transfusions. In two (4%) cases, a sternal intraosseous device failed, and transfusion was delayed. In three (6%) other cases, the team experienced missing transfusion sets, difficulties in establishing intravenous access or pain during initial transfusion.

### Out of hospital and in-hospital transfusions

Of 52 (100%) patients, 48 (92%) received LTOWB, nine (17%) received PRBC, and nine (17%) received FDP. Twenty-eight (54%) patients were transfused with one unit of LTOWB, and 20 (39%) patients were transfused with two units. Four (8%) interhospital transfers with ruptured aortic aneurysms (3) or intraabdominal bleeding and pelvic fracture (1) received 4–7 units of PRBC and 2–6 units of FDP in addition to two LTOWB units during transfer to the regional trauma centre.

Of the forty-six (88%) patients who were admitted alive to the hospital, 35 (76%) received additional transfusions of whole blood or blood components during the first 24 h; 15 (33%) received LTOWB, 31 (67%) received PRBC, 28 (61%) received thawed fresh frozen plasma (FFP), and 14 (30%) received platelet concentrates. Eleven (24%) patients were not transfused with additional blood products after admission. Out of hospital and in-hospital fluid resuscitation is shown in Table 4. Thirty-three (63%) patients received 1 g of tranexamic acid out of hospital. In the last two years of the study period, calcium chloride was added to the inventory, and four (8%) patients received 5 mmol out of hospital.

**Table 4:** Out of hospital and in-hospital volume replacement

Variable	Number of patients (%)	Volume replacement
*Out of hospital*		
Clear fluids, ml	9 (17)	716 (606)
Red blood cells, units	9 (17)	3.33 (2.35)
Plasma, units	9 (17)	1.78 (1.64)
Whole blood, units	48 (92)	1.42 (0.51)
*In-hospital,* < *24 h*		
Clear fluids, ml	37 (90)	2007 (1345)
Red blood cells, units	28 (67)	7.39 (11.99)
Plasma, units	25 (60)	8.36 (14.60)
Platelets, units	13 (32)	3.36 (5.05)
Whole blood, units	14 (34)	7.60 (7.89)

Data are presented as the number of patients (percentage) or mean (standard deviation)

### Key vital signs

**Fig. 2:**
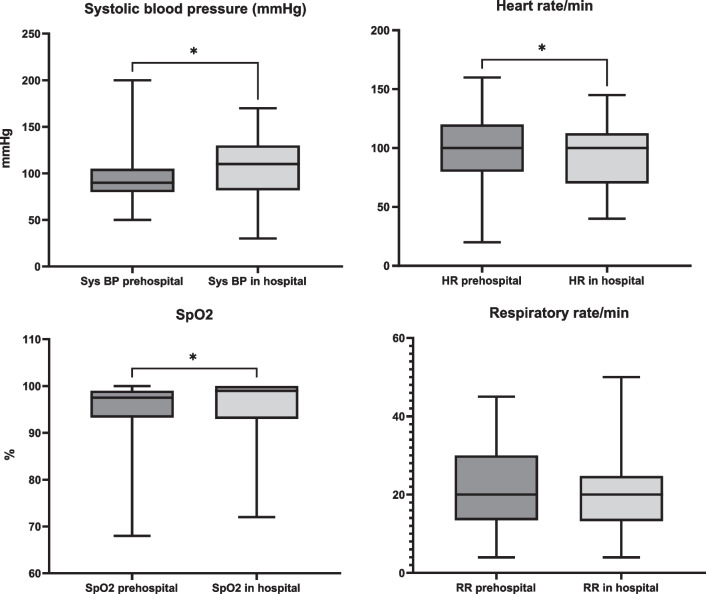
The box-and-whisker plots give out of hospital and early in-hospital key vital signs. Out of hospital and in-hospital (emergency department) values were compared using the Wilcoxon matched-pairs signed rank test, and significant *p* values were found for systolic blood pressure (SBP) (*p* = 0.0169), heart rate (*p* = 0.0169), and SpO_2_ (*p* = 0.0242). Patients with cardiac arrest were excluded from the analysis

Of the 42 non-CA patients, 24 (57%) had documented blood pressure prior to the start of transfusion. In the non-measured group, three (7%) presented with a palpable peripheral pulse, and ten (24%) presented with a palpable carotid pulse. There were no documented assessment of blood pressure or pulse in five (12%) patients. There were missing data for out of hospital SBP, respiratory rate, and pulse oximetry (SpO_2_) in 42%, 10%, and 43% of patients, respectively. Out of hospital and in-hospital vital signs are described (Fig. 2), and significant differences were found for systolic blood pressure, heart rate, and SpO_2_.

### Airway management

All 10 (19%) patients presenting with CA received advanced airway management. Of these, six (60%) received endotracheal intubation (ETI), three (30%) received a supraglottic airway device, and one (10%) received bag-mask-ventilation. Excluding CA patients, nine (21%) received rapid sequence induction and ETI, three (7%) received bag-mask ventilation, 17 (40%) received supplemental oxygen on a nonrebreather mask, and 13 (31%) received no advanced airway interventions.

### Response and transport times

The response, on-scene and transport times are presented in Table 1. The median response time was 20 min (IQR 12–35) on primary missions, 37 min (IQR 20–43) on secondary missions, and 21 min on cardiac arrest missions (IQR 10–43). The median “on-scene-time” was 7.5 min (IQR 4–19), 15 min (IQR 9–24), and 14 min (IQR 3–28), respectively. The median transport time was 19 min for primary missions (IQR 12–26), 35 min for secondary missions (IQR 20–41), and 20 min for cardiac arrest missions (IQR 12–38).

### Implementation process

Prior to implementation of the LTOWB transfusion program, over 30 units were subjected to extensive in vitro quality control analysis. As LTOWB is not an ordinary blood product in Norway, we applied to the appropriate regulatory body and were granted permission to use LTOWB in out of hospital emergencies. For the first two years of the program, the returned LTOWB units were subjected to internal quality controls (e.g., haemolysis at the end of storage) and then discarded. In December 2017, usage of LTOWB was implemented for in-hospital massive transfusion protocols. Subsequently, all returned LTOWB units were incorporated into the blood bank inventory for in-hospital use or stored for 21 days.

### Emergency interventions

In 41 of 46 admitted patients, emergency surgery (e.g., thoracotomy, laparotomy, or craniotomy) or other haemostatic interventions (e.g., sutures of skin or soft tissue lacerations, radiological interventions, bone fracture repairs and gastroscopies) were performed within the first 24 h (Table 1).

## Discussion

To our knowledge, this is the first study describing an out of hospital transfusion program with whole blood to patients in haemorrhagic shock by civilian physician-staffed HEMS in Europe. We found that the majority of exsanguination deaths occurred early and within 6 h. Overall, one in three patients receiving transfusion died within 24 h, and only one in two patients survived beyond 30 days, emphasizing the life-threatening nature of their injury or illness and the need to improve out of hospital treatment of civilian patients suffering noncompressible haemorrhage. Our results indicate a restrictive transfusion practice with an overall transfusion rate of 1.4%, comparable to transfusion rates in other air ambulance services (UK) that show approximately 3% in trauma patients receiving PRBC [15]. We found no serious complications or adverse events with whole blood during transfusion or transit. Although logistics around forward storage and transfusion of blood products in the out of hospital setting may be challenging, no adverse or logistical issues were reported. The demographics in our study are comparable with other studies describing out of hospital use of blood products in civilian trauma services, with middle-aged male patients and blunt trauma mechanisms dominating; however, our results cannot compare directly with those from studies describing military use of blood products in younger patients with combat injuries [2, 16].

The main priorities in haemostatic resuscitation are, nonetheless, to stop compressible bleeding, maintain vital organ perfusion and support haemostasis [17]. Although overall trauma mortality has decreased over the last decades due to improved trauma services and hospital infrastructure, most early deaths due to haemorrhage still occur in the pre-surgical phase [18, 19]. The use of out of hospital blood products has evolved to improve the survival of haemorrhaging patients in the field, both civilian and military [10].

Military use of whole blood has become a key element in RDCR, due to simpler logistics, reduced time to transfusion, and improved survival in combat causalities [5, 20]. Whole blood may have better global haemostasis effects, a reduced incidence of transfusion hypocalcaemia and haemodilution, and a reduction in overall blood use compared to components with higher citrate and additives contents [4, 21]. A growing body of evidence supports the use of whole blood for civilian out of hospital patients with noncompressible bleeding [9, 22]. Learning from military experiences, some civilian ambulance services (USA) have recently implemented whole blood programs with success, while the majority still deploy traditional PRBC, FDP or crystalloids [23, 24]. A recent review of air ambulance services (UK) showed that less than half of the services deployed with blood products (PRBC or FFP) [15].

For haemorrhaging patients in extremis to survive, they should have access to modern haemorrhage control treatments available in trauma hospitals as well as balanced blood transfusions [10, 25]. Tailored treatment offering out of hospital whole blood to patients with life-threatening noncompressible haemorrhage may offer a more targeted approach than traditional transfusion protocols [9, 26]. The decision to initiate transfusion in a patient with suspected haemorrhagic shock is both complex and time-critical and is based on a variety of symptoms, clinical presentations and mechanisms of injury. In patients presenting with unstable physiology or clinical signs on scene, a certain degree of overtransfusion may be inevitable [27]. Although increased survival to hospital in trauma patients receiving out of hospital transfusion of PRBC has been shown, this does not necessarily translate into increased long-term survival [28].

Out of hospital resuscitative interventions are often initiated before the cause of hypotension or hypoxia is clearly identified [29]. In our study, SBP was not consistently documented prior to resuscitation, as only approximately half of the patients had a blood pressure reading prior to transfusion. Hence, SBP was not used as a transfusion trigger in the majority of the patients. In exsanguinating patients, establishing vascular access by intraosseous or intravenous routes to enable transfusion is just one of many timely interventions that must be provided [30]. Therefore, having to prioritize resuscitative efforts before measurements may indicate the degree of haemodynamic instability in these patients and the haste involved in correcting circulatory failure [31]. As the pathophysiology behind hypotension is diverse, it is important that vital signs are interpreted along with clinical symptoms and mechanism of injury or illness by competent providers’ on-scene [32]. Having experienced physicians in the HEMS teams may ensure that life-saving interventions can be performed to the same standards as in-hospital emergency treatment without compromising on-scene times [33].

Drug-assisted rapid sequence induction and intubation is the definitive method of securing the airway in trauma patients [34]. The decision whether to intubate patients in haemorrhagic shock may be challenging. The timing of intubation is crucial, and despite current European guidelines, ETI on-scene is not always done [35]. ETI should be restricted to those showing signs of increasing airway compromise or deteriorating level of consciousness [25]. Detrimental effects of advanced airway management strategies in exsanguinating patients have been reported [36]. The physiological effects of ETI and subsequent positive pressure ventilation on venous return may mimic the physiology of tension pneumothorax in hypovolemic patients, leading to a further decrease in cardiac output [37, 38]. Therefore, in patients with noncompressible haemorrhage, reducing on-scene times and postponing ETI until haemorrhage control is achieved may increase survival [25]. Knowledge of whether, when and how to intubate these patients is important [39].

An out of hospital balanced transfusion approach with components is challenging due to different storage conditions for PRBCs, plasma and platelets. Whole blood represents an alternative that is logistically superior and easier to handle in time-critical emergencies with short on-scene and flight times compared to blood components [20]. The production of a leukocyte-reduced, platelet-containing LTOWB unit in our blood bank also requires fewer logistical steps compared to a unit of PRBC concentrate. This whole blood fulfils all quality control parameters detailed in national requirements as well as the European Guidelines concerning testing for infectious agents, haemolysis, and leukocyte depletion [14]. Our system, where unused units are returned and incorporated into the blood bank inventory for further in-hospital use, minimizes potential waste.

The strength of our study is the prospective design involving experienced physicians in charge of patient treatment and data registration and the inclusion of 52 patients in haemorrhagic shock receiving out of hospital blood transfusions over a 5-year study period. The limitations are the small number of patients reflecting the low frequency of haemorrhagic shock in our area of operations and the selection bias represented by the excluded patients. Our patient cohort was also a heterogeneous mix of trauma and nontrauma patients, with different mechanisms of injuries/illnesses and high mortality rates. The ethical committee required written informed consent from all surviving participants. Unfortunately, this was not possible to achieve in sixteen patients. The excluded patients did not differ significantly from the included patients regarding demographics or mechanisms of injury/illness, and we found the study patients to be representative of the target population. Therefore, this selection bias may be of less importance in regard to interpreting our results.

Patients in extremis who require immediate out of hospital blood transfusions are rare, but prospective observational trials can contribute to the overall evidence in this field [40]. More studies are needed to clarify the role of whole blood in civilian out of hospital haemostatic resuscitation and whether early transfusions reduce the overall use of blood products in these patients, as well as aid in developing more objective transfusion protocols. The experience and research gained from the implementation of advanced interventions, such as whole blood transfusion programs in HEMS, may provide guidance on future resuscitation strategies for these patients [25].

## Conclusions

Our results indicate that implementing a whole blood transfusion program in civilian HEMS is feasible and safe and that the logistics around out of hospital whole blood transfusions are manageable.

## Supplementary Information

**Additional file 1**. Patient NACA score.

## Data Availability

All data analysed during this study are included in this published article and its supplementary information files.

## Abbreviations

RDCR: Remote damage control resuscitation
PRBC: Packed red blood cells
HEMS: Helicopter emergency medical service
LTOWB: Low-titre Group O whole blood
SBP: Systolic blood pressure
GCS: Glasgow Coma Scale
NACA: The National Advisory Committee on Aeronautics severity score
FDP: Freeze-dried plasma
IQR: Interquartile ranges
CA: Cardiac arrest
FFP: Fresh frozen plasma
SpO_2_: Pulse oximetry
ETI: Endotracheal intubation

## References

[1] McCullough AL, Haycock JC, Forward DP, Moran CG (2014). Major trauma networks in England. Br J Anaesth.

[2] Crombie N, Doughty HA, Bishop JRB, Desai A, Dixon EF, Hancox JM (2022). Resuscitation with blood products in patients with trauma-related haemorrhagic shock receiving prehospital care (RePHILL): a multicentre, open-label, randomised, controlled, phase 3 trial. Lancet Haematol.

[3] Woolley T, Round JA, Ingram M (2017). Global lessons: developing military trauma care and lessons for civilian practice. Br J Anaesth.

[4] Spinella PC, Gurney J, Yazer MH (2019). Low titer group O whole blood for prehospital hemorrhagic shock: it is an offer we cannot refuse. Transfusion.

[5] Shackelford SA, Del Junco DJ, Powell-Dunford N, Mazuchowski EL, Howard JT, Kotwal RS (2017). Association of prehospital blood product transfusion during medical evacuation of combat casualties in afghanistan with acute and 30-day survival. JAMA.

[6] Bodnar D, Rashford S, Williams S, Enraght-Moony E, Parker L, Clarke B (2014). The feasibility of civilian prehospital trauma teams carrying and administering packed red blood cells. Emerg Med J.

[7] Bjerkvig C, Sivertsen J, Braathen H, Lunde THF, Strandenes G, Assmus J (2020). Cold-stored whole blood in a Norwegian emergency helicopter service: an observational study on storage conditions and product quality. Transfusion.

[8] Seheult JN, Bahr M, Anto V, Alarcon LH, Corcos A, Sperry JL (2018). Safety profile of uncrossmatched, cold-stored, low-titer, group O+ whole blood in civilian trauma patients. Transfusion.

[9] Hazelton JP, Ssentongo AE, Oh JS, Ssentongo P, Seamon MJ, Byrne JP (2022). Use of cold-stored whole blood is associated with improved mortality in hemostatic resuscitation of major bleeding: a multicenter study. Ann Surg.

[10] Rangrass G (2022). Whole blood use in trauma resuscitation: targeting prehospital transfusion. Curr Opin Anaesthesiol.

[11] Raatiniemi L, Mikkelsen K, Fredriksen K, Wisborg T (2013). Do pre-hospital anaesthesiologists reliably predict mortality using the NACA severity score? A retrospective cohort study. Acta Anaesthesiol Scand.

[12] von Elm E, Altman DG, Egger M, Pocock SJ, Gotzsche PC, Vandenbroucke JP (2007). Strengthening the Reporting of Observational Studies in Epidemiology (STROBE) statement: guidelines for reporting observational studies. BMJ.

[13] Hagen KG, Strandenes G, Kristoffersen EK, Braathen H, Sivertsen J, Bjerkvig CK (2021). A whole blood based resuscitation strategy in civilian medical services: experience from a Norwegian hospital in the period 2017–2020. Transfusion.

[14] European Directorate for the Quality of Medicines & HealthCare (EDQM CoE). Guide to the preparation, use and quality assurance of blood components. https://www.edqm.eu/en/blood-transfusion-guide (2017).

[15] Naumann DN, Hancox JM, Raitt J, Smith IM, Crombie N, Doughty H (2018). What fluids are given during air ambulance treatment of patients with trauma in the UK, and what might this mean for the future? Results from the RESCUER observational cohort study. BMJ Open.

[16] Gurney J, Staudt A, Cap A, Shackelford S, Mann-Salinas E, Le T (2020). Improved survival in critically injured combat casualties treated with fresh whole blood by forward surgical teams in Afghanistan. Transfusion.

[17] Kovacs G, Sowers N (2018). Airway management in Trauma. Emerg Med Clin N Am.

[18] de Knegt C, Meylaerts SA, Leenen LP (2008). Applicability of the trimodal distribution of trauma deaths in a Level I trauma centre in the Netherlands with a population of mainly blunt trauma. Injury.

[19] Chalkley D, Cheung G, Walsh M, Tai N (2011). Deaths from trauma in London–a single centre experience. Emerg Med J.

[20] Levin D, Zur M, Shinar E, Moshe T, Tsur AM, Nadler R (2021). Low-titer group o whole-blood resuscitation in the prehospital setting in Israel: review of the first 2.5 years' experience. Transfus Med Hemother.

[21] Kornblith LZ, Howard BM, Cheung CK, Dayter Y, Pandey S, Busch MP (2014). The whole is greater than the sum of its parts: hemostatic profiles of whole blood variants. J Trauma Acute Care Surg.

[22] Braverman MA, Smith A, Pokorny D, Axtman B, Shahan CP, Barry L (2021). Prehospital whole blood reduces early mortality in patients with hemorrhagic shock. Transfusion.

[23] Brill JB, Tang B, Hatton G, Mueck KM, McCoy CC, Kao LS (2022). Impact of incorporating whole blood into hemorrhagic shock resuscitation: analysis of 1,377 consecutive trauma patients receiving emergency-release uncrossmatched blood products. J Am Coll Surg.

[24] Selleng K, Baschin M, Henkel B, Jenichen G, Thies KC, Rudolph M (2021). Blood product supply for a helicopter emergency medical service. Transfus Med Hemother.

[25] Ter Avest E, Carenzo L, Lendrum RA, Christian MD, Lyon RM, Coniglio C (2022). Advanced interventions in the pre-hospital resuscitation of patients with non-compressible haemorrhage after penetrating injuries. Crit Care.

[26] Hofer S, Schlimp CJ, Casu S, Grouzi E (2021). Management of coagulopathy in bleeding patients. J Clin Med.

[27] Gavrilovski M, Griggs JE, Ter Avest E, Lyon RM (2021). The contribution of helicopter emergency medical services in the pre-hospital care of penetrating torso injuries in a semi-rural setting. Scand J Trauma Resusc Emerg Med.

[28] Rehn M, Weaver A, Brohi K, Eshelby S, Green L, Roislien J (2018). Effect of pre-hospital red blood cell transfusion on mortality and time of death in civilian trauma patients. Shock.

[29] Vincent JL, De Backer D (2013). Circulatory shock. N Engl J Med.

[30] Sorgjerd R, Sunde GA, Heltne JK (2019). Comparison of two different intraosseous access methods in a physician-staffed helicopter emergency medical service: a quality assurance study. Scand J Trauma Resusc Emerg Med.

[31] Bjerkvig CK, Strandenes G, Eliassen HS, Spinella PC, Fosse TK, Cap AP (2016). "Blood failure" time to view blood as an organ: how oxygen debt contributes to blood failure and its implications for remote damage control resuscitation. Transfusion.

[32] Seymour CW, Cooke CR, Heckbert SR, Copass MK, Yealy DM, Spertus JA (2013). Prehospital systolic blood pressure thresholds: a community-based outcomes study. Acad Emerg Med.

[33] Lockey DJ, Crewdson K, Davies G, Jenkins B, Klein J, Laird C (2017). AAGBI: safer pre-hospital anaesthesia 2017: Association of Anaesthetists of Great Britain and Ireland. Anaesthesia.

[34] NICE. Major trauma: assessment and initial management [NG39]. https://www.nice.org.uk/guidance/ng39 (2016). 26913320

[35] Stausberg T, Ahnert T, Thouet B, Lefering R, Bohmer A, Brockamp T (2022). Endotracheal intubation in trauma patients with isolated shock: universally recommended but rarely performed. Eur J Trauma Emerg Surg Off Publ Eur Trauma Soc.

[36] Crewdson K, Rehn M, Brohi K, Lockey DJ (2018). Pre-hospital emergency anaesthesia in awake hypotensive trauma patients: beneficial or detrimental?. Acta Anaesthesiol Scand.

[37] Funk DJ, Jacobsohn E, Kumar A (2013). Role of the venous return in critical illness and shock: part II-shock and mechanical ventilation. Crit Care Med.

[38] Shafi S, Gentilello L (2005). Pre-hospital endotracheal intubation and positive pressure ventilation is associated with hypotension and decreased survival in hypovolemic trauma patients: an analysis of the National Trauma Data Bank. J Trauma.

[39] Lockey DJ, Crewdson K, Lossius HM (2014). Pre-hospital anaesthesia: the same but different. Br J Anaesth.

[40] Shea SM, Staudt AM, Thomas KA, Schuerer D, Mielke JE, Folkerts D (2020). The use of low-titer group O whole blood is independently associated with improved survival compared to component therapy in adults with severe traumatic hemorrhage. Transfusion.

