# Supplementary material for: Implementation of a low-titre whole blood transfusion program in a civilian helicopter emergency medical service
Source: Scand J Trauma Resusc Emerg Med. 2022 Dec 9;30:65. doi: 10.1186/s13049-022-01051-z (PMC9733220; doi:10.1186/s13049-022-01051-z)
Supplement: Supplementary file 1 — Additional file 1. Patient NACA score. [file 13049_2022_1051_MOESM1_ESM.docx]

#
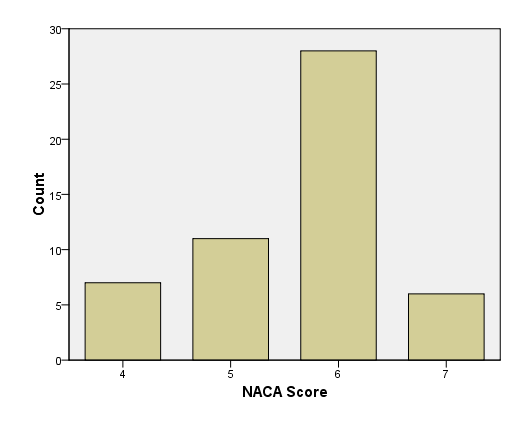
Additional file 1: Patient NACA score

The severity scoring used to classify injury or illness severity in the Norwegian National Air Ambulance Service as originally described by the National Advisory Committee on Aeronautics (NACA).

NACA 0 - No injury or disease

NACA l - Injuries/diseases without any need for acute physician care

NACA 2 - Injuries/diseases requiring examination and therapy by a physician, but hospital admission is not indicated

NACA 3 - Injuries/diseases without acute threat to life but requiring hospital admission

NACA 4 - Injuries/diseases that can possibly lead to deterioration of vital signs

NACA 5 - Injuries/diseases with acute threat to life

NACA 6 - Injuries/diseases transported after successful resuscitation of vital signs

NACA 7 - Lethal injuries or diseases (with or without resuscitation attempts)
